# Reference norms for evaluating maximum expiratory flow of children and adolescents of the Maule Region in Chile

**DOI:** 10.7717/peerj.5157

**Published:** 2018-07-16

**Authors:** Marco Antonio Cossio-Bolaños, Cynthia Andruske, Miguel Arruda, Jose Sulla-Torres, Jaime Pacheco-Carrillo, Camilo Urra-Albornoz, Rossana Gomez-Campos

**Affiliations:** 1 Departamento de Ciencias de la Actividad Física, Universidad Católica del Maule, Talca, Chile; 2 Faculty of Physical Education, State University of Campinas, Campinas, São Paulo, Brazil; 3 Universidad Nacional de San Agustin, Arequipa, Perú; 4 Centro de Investigación Especializado en Ciencias de la Educación, Salud y Deporte, CINEMAROS, Arequipa, Perú; 5 Universidad Católica de Santa María, Arequipa, Perú; 6 Universidad del Bio Bio, Chillan, Chile; 7 Facultad de Salud, Carrera de Kinesiología, Universidad Santo Tomás, Talca, Chile; 8 Universidad Autonoma de Chile, Talca, Chile

**Keywords:** Maximum expiratory flow, Biological age, Chile, Percentiles

## Abstract

**Background:**

The norms for evaluating the maximum expiratory flow (MEF) usually are developed according to chronological age and height. However, to date, little research has been conducted using reference values that take into account the temporal changes of biological maturation. The objectives of this study were to (a) compare the MEF with those of other international studies, (b) align the MEF values with chronological and biological age, and (c) propose reference standards for children and adolescents.

**Methods:**

The sample studied consisted of 3,566 students of both sexes (1,933 males and 1,633 females) ranging in age from 5.0 to 17.9 years old. Weight, standing height, and sitting height were measured. Body mass index was calculated. Biological maturation was predicted by using age of peak height velocity growth (APHV). MEF (L/min) was obtained by using a forced expiratory manoeuvre. Percentiles were calculated using the LMS method.

**Results and Discussion:**

Predicted APHV was at age 14.77 ± 0.78 years for males and for females at age 12.74 ± 1.0 years. Biological age was more useful than chronological age for assessing MEF in both sexes. Based on these findings, regional percentiles were created to diagnose and monitor the risk of asthma and the general expiratory status of paediatric populations.

## Introduction

Spirometry is a method used to measure the pulmonary function of children, adolescents, and adults (Beydon et al., 2007). This method is used to detect changes in lung function, and it is necessary for measuring performance and tracking respiratory illnesses (García-Río et al., 2013; Jiang et al., 2015).

The most common spirometric parameters include the forced vital capacity, vital capacity, maximum volume of air exhaled during the first second of the forced breath (FEV1), and the maximum expiratory flow (MEF). MEF is defined as the maximum flow of air exhaled during a forced breath initiated during maximum pulmonary inflation (Kano et al., 1993). Furthermore, it is a useful indicator for predicting the risk of asthma monitoring the respiratory state of obese and non-obese children and adolescent students (Arets, Brackel & van der Ent, 2001; Dixit, Raje & Agrawal, 2005).

In general, the MEF is influenced by age, body surface, obesity, physical activity, posture, environment, altitude, tobacco consumption, and racial differences (Siregar et al., 2009) and in minor proportions socio-economic factors (Raju et al., 2005).

In this context, for the diagnosis, classification, and tracking of the MEF in children and adolescents, it is necessary to have specific reference values for a population. Based on the current research literature, various studies exist that have developed regression equations to determine the MEF of children and adolescents (Knudson et al., 1983; Facchini et al., 2007; Gutiérrez et al., 2014). In different countries around the world, other researchers have proposed percentiles based on age, height, and sex (Cobos-Barroso, Reverté-Bover & Liñán-Cortés, 1996; Bianchi & Baiardi, 2008). These values rise as age increases.

Thus, the mathematical models proposed to predict MEF and generated based on chronological age and anthropometric variables could bias the results because biological age is determined when studying populations during the growth process and development. Also, added to this is that the proposed reference values, in general, are derived from small samples (Stocks & Quanjer, 1995), and they do not cover the total pediatric range (Merkus et al., 2010). In many cases, due to the secular trend, the reference values need to be updated.

Based on an extensive review of international and national research literature, we discovered that no studies exist hat show evidence of reference values that take into account temporal growth changes and biological maturation. Experts acknowledge that the variation between individuals of the same biological age during puberty are great (Iuliano-Burns, Mirwald & Bailey, 2001). Therefore, even to a large extent, inspiratory muscle strength may depend on biological maturation.

Consequently, the authors of this study hypothesize that biological age could be more useful when evaluating the MEF in relation to chronological age. In addition, the percentiles could be used to classify into categories the MEF for both children and adolescents of both sexes from the Maule Region (Chile).

As a result of the previous challenges, this study was designed to include a representative sample. In addition, biological age was controlled for. Furthermore, the LMS (L: lambda, skewness, M: median, and S: sigma; co-efficient of variation) statistical method was used in order to control for the extremes of the percentiles (Kułaga et al., 2011).

Two objectives guided this research. They included the following: (a) to align the values of MEF by chronological and biological age and (b) to propose reference standards of MEF for children and adolescents of the Maule Region, Chile.

## Methods

### Design and subjects

A cross-sectional descriptive study was carried out. The sample population studied consisted of 25,471 students of both sexes (ages ranging from 5.0 to 17.9 years). The sample was selected through stratified random sampling (probabilistic). The resulting representative sample consisted of 14% of the population (3,566 students of both sexes): 1,933 males (7.6%) and 1,633 females (6.4%). All of the students were selected from 12 public schools from elementary and secondary from the public education system from the Maule Region (Chile). Predicted age of peak height velocity (APHV) was at age 14.77 ± 0.78 years for males and for females at age 12.74 ± 1.0 years.

Talca, the research site, is the capital of the Maule Region (Chile). It is located 243 km south of Santiago (the capital of Chile). This region is considered to be an agricultural, economic, and cultural center for the region. Based on these characteristics, it is regarded as the most important central valley of Chile. The 2012 Human Development Index for the Maule Region was 0.72.

Parents were informed about the objectives of the study. At that time, the variables that were to be collected were also described to the parents or guardians. Each parent or guardian was given a copy of the informed consent form to sign in order to give permission for their children to participate in the study. Children and adolescents returning the consent forms (signed by the responsible parent or guardian) were visited in their respective schools by the researchers responsible for the project. The visits took place during classes from 8:00 a.m. to 11:00 a.m. Monday to Friday during the months of August to November during 2015.

Parents provided information about any respiratory illnesses or health issues occurring during the last three weeks before the study began, for example, signs of asthma, a persistent fever, pneumonia, and allergic rhinitis. As a result, we excluded from the research study, children and adolescents with any types of the health issues described above or whose parents smoked.

The experimental protocol was in accord with the Helsinki Declaration (World Health Organization for Human Subjects). Furthermore, the study complied with the consent and permissions from the respective school administrations and the Ethics Committee from the Universidad Autónoma de Chile, Talca, Chile, UA 238/2014.

## Procedures and Measures

Student information, such as age, sex, and home address, was collected from the administration of the 12 schools selected for the study. Ages were grouped into 13 categories from age 5.0 to 17.9 years old. Intervals by age were distributed in the following way: 5.0–5.9 years, for example.

The disease history of students for the respiratory tract and smoking habits were obtained from a simple questionnaire that was attached to the informed consent form. Anthropometric variables and MEF were evaluated by four health professionals (authors of this article).

The anthropometric variables of weight, standing height, and sitting height were evaluated according to the protocol described by Ross & Marfell-Jones (1991). Body weight (kg) was measured using an electric scale (Tanita, Glasgow, United Kingdom, Ltd.) with a scale of 0–150 kg and a precision of 100 g. Standing height was measured with a portable stadiometer (Seca & Co. KG, Hamburg, Germany) with an accuracy of 0.1 mm and a scale of 0–2.50 m. When both of the variables were measured, students were barefoot and wearing only shorts and a short shirt. Sitting height was taken using a wooden bench 50 cm high with a measurement scale of 0–150 cm with a precision of one mm. Body mass index (BMI) was calculated using the formula proposed by Quetelet (BMI = weight (kg)/height (m^2^)).

The measurement of the MEF (L/min) was measured by using a Mini Wright device (Clement Clarke International Ltd., Essex, England) with a range of 60–900 L/min. The MEF was obtained by a forced expiratory manoeuver commencing with a maximum inhalation (equal to that as in spirometry). Following the suggestions of Quanjer et al. (1993), the measurement was taken while the individual was standing without flexing the neck. Prior to the measurement, the students were informed about the use of the device. Before being evaluated, adolescents engaged in three practice attempts (familiarization). The students were instructed about the technique of the manoeuvers to carry out (forced inhalation followed by a rapid exhalation). Students performed three attempts, and the highest value was recorded.

To guarantee the quality control of the anthropometric variables and the MEF, 10% (356 subjects) of the sample was evaluated twice. The technical error of measurement intra and inter-evaluator for anthropometry showed values between 1% and 2% and for the MEF between 2% and 3.5%.

Biological maturation (somatic maturation) was determined by means of the regression equation proposed by Mirwald et al. (2002). This technique indicates the time before or after APHV. To estimate the APHV, multiple regression equations by sex were used. Standing height, sitting height, leg length (standing height–sitting height), decimal age, and their interactions were also included. Biological age was constructed using intervals of 1 year, represented in males from −6 APHV to 3 APHV and in females from −6 APHV to 5 APHV.

### Statistics

The normal distribution of the data was verified by means of the Kolmogorov–Smirnov test. The analysis was carried out through descriptive statistical analysis of the arithmetic mean and the standard deviation. Differences between genders were determined by the test for independent samples. Smoothed percentile curves were created for the MEF for each sex based on the LMS method (Cole et al., 2009). LMS Chart Maker Pro Version 2.3 software (Pan & Cole, 2006) was used. The final percentile curves were the result of smoothing three age-specific curves. L (lambda; skewness), M (Mu; median), and S (sigma; co-efficient of variation). Percentiles p5, p10, p15, p25, p50, p85, p90, and p95 were created. The level of significance adopted was 0.001. Calculations were made by using EXCEL sheets and SPSS 16.0.

## Results

The anthropometric profile of the sample studied is illustrated in Table 1. Males showed greater weight and standing height than females commencing from age 14 to 17 years (*p* < 0.001). Furthermore, the males’ sitting height was greater than that of the females from age 15 to 17 years (*p* < 0.001). No significant differences were found in the BMI between the sexes except at age 13 where the females showed a higher value than the males (*p* < 0.001). In general, all of the variables increased as age advanced.

**Table 1:** Anthropometric characteristics of the sample studied.

Ages (years)	*N*	Weight (Kg)		Standing height (cm)		Sitting height (cm)		BMI (Kg/m^2^)	
		*X*	SD	*X*	SD	*X*	SD	*X*	SD
**Males**									
5.0–5.9	48	22.2	4.5	115.7	5.8	61.6	2.5	17.1	3.1
6.0–6.9	55	24.9	10.9	124.1	12.2	64.4	6.6	17.8	3.3
7.0–7.9	54	30.9	7.5	127.4	7.9	66.7	4.0	18.8	3.1
8.0–8.9	46	31.1	5.9	129.8	4.8	68.6	3.5	18.4	2.8
9.0–9.9	52	37.6	10.3	138.0	7.0	71.2	4.1	19.6	4.6
10.0–10.9	74	42.5	9.6	144.8	8.3	75.9	4.4	20.2	3.7
11.0–11.9	196	48.1	10.3	149.3	7.1	78.1	4.9	21.5	3.7
12.0–12.9	268	52.4	11.1	156.6	8.3	82.3	4.8	21.3	4.0
13.0–13.9	272	55.6	11.0	161.7	8.1	84.3	5.1	21.2	3.3
14.0–14.9	242	63.5	12.9*	167.0*	8.0	87.0	7.3	22.7	4.2
15.0–15.9	313	64.8	11.6*	169.3*	7.8	88.2	6.3*	22.6	4.2
16.0–16.9	201	71.3	13.5*	172.9*	6.6	89.6	6.5*	23.8	4.1
17.0–17.9	112	72.8	12.0*	174.4*	7.9	90.2	5.2*	23.8	4.7
**Females**									
5.0–5.9	50	22.1	4.2	117.2	2.3	60.2	3.1	16.1	4.1
6.0–6.9	53	23.3	4.4	118.4	5.9	61.4	3.0	16.5	2.5
7.0–7.9	59	28.5	4.1	124.0	4.0	65.6	2.9	18.5	1.9
8.0–8.9	56	31.8	5.3	133.1	1.8	69.4	2.6	17.9	2.8
9.0–9.9	68	34.6	6.0	133.6	6.4	70.7	4.8	19.3	2.1
10.0–10.9	74	42.4	8.7	142.9	7.4	76.7	4.9	20.6	2.9
11.0–11.9	148	47.7	10.2	149.9	7.0	79.1	5.0	21.1	3.8
12.0–12.9	319	51.7	11.1	154.8	5.8	82.3	5.3	21.5	4.0
13.0–13.9	237	56.3	11.7	156.2	5.9	83.3	6.3	23.0	4.3*
14.0–14.9	182	58.9	12.9	159.2	6.6	85.5	3.3	23.2	4.7
15.0–15.9	162	60.2	11.2	159.6	5.9	86.0	3.2	23.5	3.6
16.0–16.9	127	60.4	11.2	159.3	5.8	85.6	3.3	23.8	4.3
17.0–17.9	98	64.3	11.9	160.2	6.5	85.7	6.3	25.1	4.3

**Notes:**

*X*, average; SD, standard deviation; BMI, body mass index.

* *p* < 0.05.

**Figure 1:**
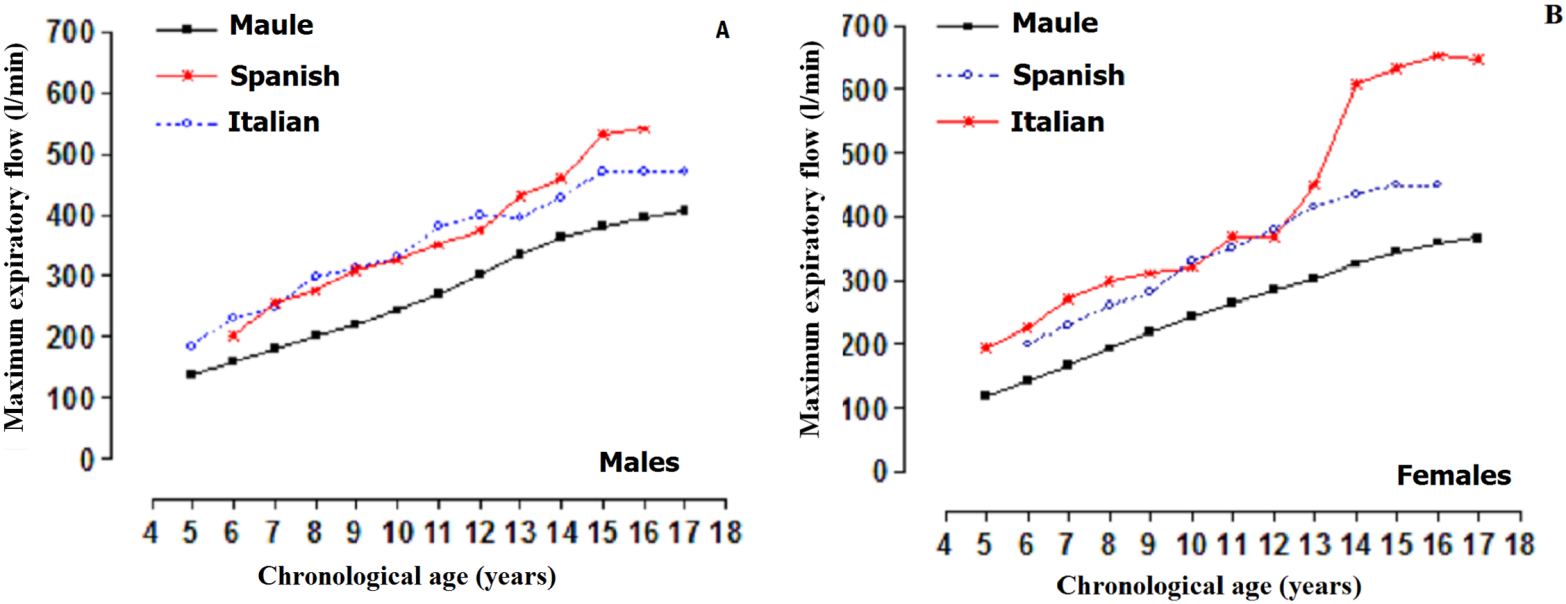
MEF of children and adolescents of the Maule Region (Chile) and international studies: Comparison between percentiles (p50). Spanish (Cobos-Barroso, Reverté-Bover & Liñán-Cortés, 1996), Italian (Bianchi & Baiardi, 2008). (A) Males and (B) Females.

Comparison of the MEF between students of the Maule Region (Chile) and those of international studies are illustrated in Fig. 1. The comparisons were carried out based on the (p50) percentile. The three studies (males and females) showed ascending values as chronological age advanced. Note that the children and adolescents of the Maule Region (Chile) showed inferior values in relation to those of the Spanish and Italian samples.

**Figure 2:**
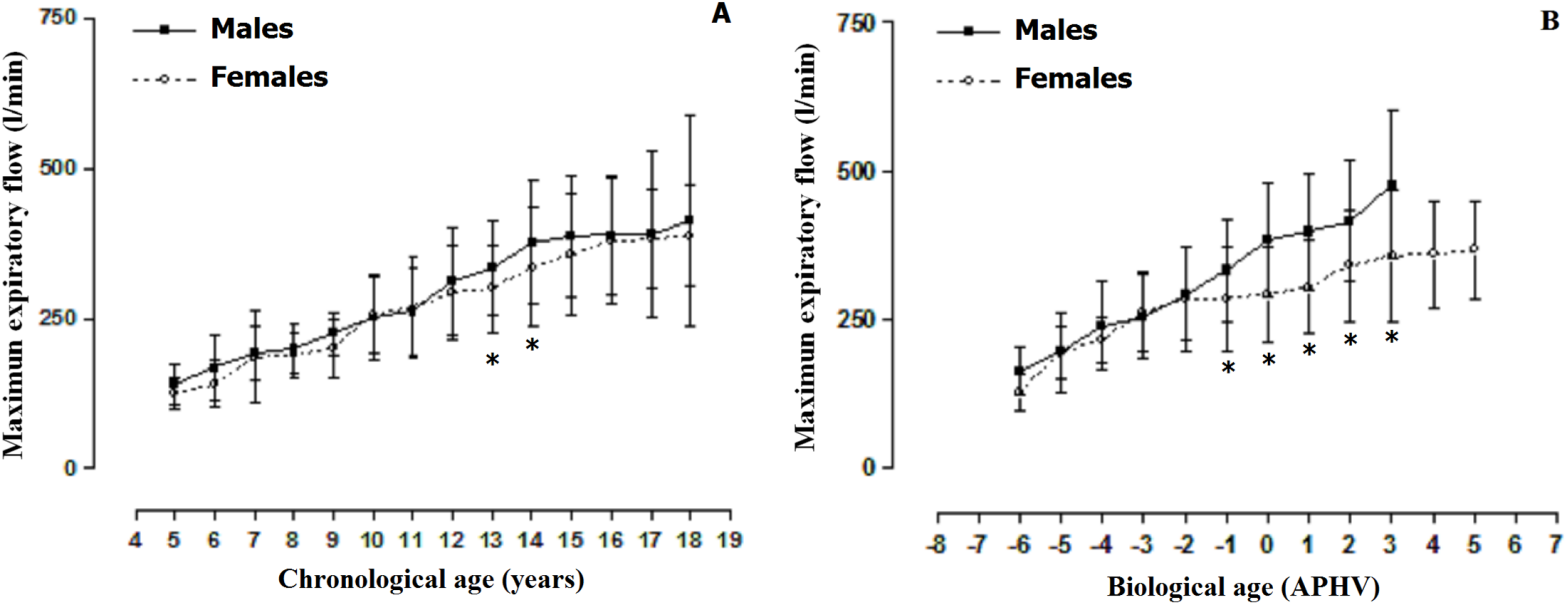
Mean values ± SD (Standard deviation) of the maximum expiratory flow arranged by chronological age (A) and biological age (B). *: significant difference in relation to men.

The comparisons of the MEF by sex can be observed in Fig. 2. When arranged by chronological age, males demonstrated greater MEF at ages 13 and 14 when compared to the females. However, at all the other ages, the values were relatively similar (*p* > 0.001). When arranged by biological age, differences by sex began to emerge commencing at −1 APHV to 5 APHV. Therefore, males showed greater MEF in relation to females, especially 1 year prior to APHV occurring.

**Figure 3:**
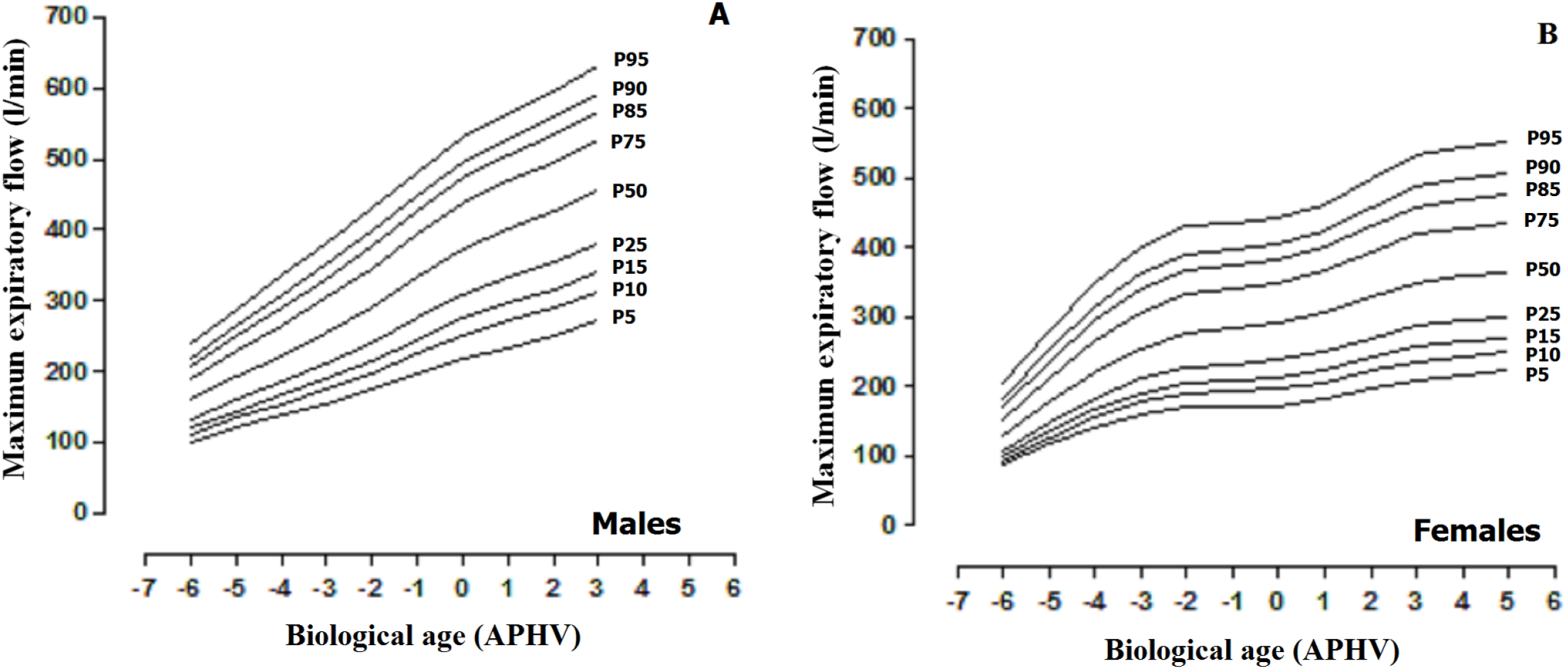
Smoothed reference curves calculated by the LMS method to assess the maximum expiratory flow (MEF) based on biological age (APHV) and sex. (A) Males; (B) Females.

The MEF percentiles based on sex and biological age are illustrated in Table 2 and Fig. 3. The values of the median in both sexes increased as the APHV advanced. The maximum values in males (MEF) were reached at 3 APHV and in females at 5 APHV.

**Table 2:** Percentile distribution of the maximum expiratory flow by gender and biological age.

APHV		L	M	S	P5	P10	P15	P25	P50	P75	P85	P90	P95
**Males**													
−6	94	0.28	159.80	0.26	101.7	113.0	121.1	133.7	159.8	189.3	206.7	219.1	238.4
−5	85	0.32	192.08	0.26	120.7	134.5	144.5	160.1	192.1	228.2	249.4	264.5	288.0
−4	73	0.37	223.78	0.27	138.6	155.3	167.2	185.8	223.8	266.4	291.2	308.7	336.0
−3	169	0.48	254.73	0.27	155.3	175.1	189.1	210.9	254.7	302.9	330.6	350.1	380.0
−2	304	0.63	291.06	0.27	174.6	198.4	215.1	240.6	291.1	345.0	375.3	396.3	428.3
−1	305	0.80	332.90	0.26	196.7	225.5	245.4	275.3	332.9	392.6	425.4	447.9	481.7
0	352	0.96	372.81	0.26	217.8	251.7	274.7	308.8	372.8	437.3	472.1	495.7	530.8
1	335	1.05	400.91	0.25	234.0	271.3	296.3	333.0	400.9	468.2	504.1	528.3	564.1
2	164	1.07	425.87	0.25	251.2	290.4	316.6	355.0	425.9	495.9	533.2	558.3	595.4
3	52	1.07	453.83	0.24	271.0	311.9	339.4	379.6	453.8	527.2	566.4	592.7	631.7
**Females**													
−6	55	−0.43	127.60	0.26	86.1	93.4	98.8	107.7	127.6	153.2	170.0	182.9	204.8
−5	64	−0.27	176.52	0.27	116.6	127.3	135.2	148.1	176.5	212.3	235.3	252.8	281.7
−4	71	−0.11	220.02	0.27	142.0	156.1	166.6	183.4	220.0	265.0	293.3	314.3	348.7
−3	79	0.04	254.47	0.28	160.4	177.8	190.5	210.8	254.5	306.7	338.8	362.3	400.1
−2	115	0.19	275.76	0.28	169.9	189.8	204.3	227.3	275.8	332.2	366.1	390.7	429.4
−1	130	0.34	283.18	0.28	171.1	192.7	208.2	232.7	283.2	340.5	374.2	398.3	435.8
0	295	0.45	289.40	0.28	172.3	195.3	211.8	237.4	289.4	347.1	380.5	404.0	440.4
1	303	0.50	304.76	0.28	180.5	205.2	222.7	249.9	304.8	365.0	399.6	423.9	461.3
2	202	0.49	329.16	0.28	195.3	221.8	240.7	270.0	329.2	394.4	431.9	458.3	498.9
3	172	0.44	349.25	0.28	208.7	236.3	256.0	286.8	349.3	418.7	458.9	487.3	531.2
4	98	0.39	357.37	0.28	216.3	243.8	263.5	294.3	357.4	428.2	469.4	498.7	544.1
5	49	0.33	362.25	0.27	222.6	249.6	269.0	299.4	362.2	433.4	475.2	505.0	551.4

**Note:**

APHV, age of peak height velocity; M, median; L, Box–Cox transformation; S, coefficient of variation, P, percentile.

## Discussion

The first objective of this study aligned the MEF values by chronological and biological age. The results showed that no significant differences occurred in both sexes when compared by chronological age except at ages 13 and 14 years. However, when arranged by biological age, males demonstrated greater MEF commencing at −1 APHV to 3 APHV. These results suggest that chronological age is of limited use for analyzing inspiratory muscle strength during childhood and adolescence.

In fact, some previous studies have indicated that during the growth stage and development of anthropometric characteristics play a fundamental role in the determination of the inspiratory muscle strength (Silva, Viana & Sousa, 2006). Therefore, during puberty, the adipose tissue predominates in girls while muscle mass increases considerably in boys (Castilho & Barras Filho, 2000). Therefore, the linear increase in the MEF values based on age, height, and weight basically depend on biological maturation.

The results of this study suggest that the MEF should be analyzed and interpreted based on biological age despite the fact that studies carried out with children and adolescents normally do not control for individual differences of sex and the state of biological maturation (Coelho-e-Silva et al., 2013). As such, the differences found in this research could be justified due to the influence of testosterone, the changing form of the thorax, and the changes in the respiratory muscles that tend to occur during puberty (Schrader, Quanjer & Olievier, 1988). Furthermore, these sex differences based on lung function are maintained during adulthood and should be taken into account during clinical research as well as during medical practice respectively (Townsend, Miller & Prakash, 2012).

With regards to comparing the findings from this research to those of international studies, the results showed values less than those students from Spain and Italy. These results could be due to ethnic factors between populations since previous studies have shown differences between student populations in diverse geographical regions around the world (Trabelsi et al., 2004; Radeos & Camargo, 2004; Alexandraki et al., 2010). In addition, they highlight that a positive relationship exists between inspiratory muscle strength, age, and height (Alexandraki et al., 2010; Gundogdu & Gundogdu, 2008).

Thus, the greater height would be related to increased levels of MEF. Consequently, this suggests that the students in this study were short for their age and reflected lower MEF values. In part, this could explain the results found in this study since the Chilean students in general were shorter in height for their age when compared to the international references (Vargas et al., 2014). However, other compromising environmental factors that might have affected the results cannot be ruled out, such as childhood health, nutritional state, environmental air quality (Ip et al., 2000), and sedentary lifestyle (Eisenmann et al., 2007).

In essence, based on the results of the first objective of the study, a second objective was developed for this study that proposed reference norms for evaluating MEF of children and adolescents based on biological age. These norms cover a wide age range from 5.0 to 17.9 years. Furthermore, they could help determine the low, normal, and/or elevated values of the inspiratory muscle strength of children and adolescents of both sexes.

Basically, the norms facilitate evaluation not only of individual patients but also of populations in general (Mellies, Stehling & Dohna-Schwake, 2014). In addition, the interpretation of the functional respiration tests (American Thoracic Society (ATS), 1991) suggested interpreting with caution borderline values.

However, the number of categories and the exact cutoff points are arbitrary and, in general, serve to evaluate the engagement of the respiratory muscles (Pellegrino et al., 2005).

In general, the ability to work and function in daily life is directly related to lung function, and both criteria should be used for qualifying the deterioration of various physiological systems (American Medical Association, 1995). In this sense, in some studies, the p10 percentile is considered to be the normal limit (Cobos-Barroso, Reverté-Bover & Liñán-Cortés, 1996; Bianchi & Baiardi, 2008). However, for this study, we adopted the p15 in order to identify percentiles with mild inspiratory muscle strength, p10 for moderate, and p5 for severe.

Thus, the norms developed here could provide additional information for health professionals. Moreover, they could help detect a real change in lung function of children and adolescents of school age. However, more studies are needed to determine with greater accuracy the threshold levels of MEF.

The Mini Wright instrument may be considered to be the oldest and simplest technique for measuring MEF. The instrument used for this study was sufficient for evaluating the lung function of children and adolescents. The techniques and procedures were safe, fast to carry out, non-invasive, economical, and applicable for field studies while providing relevant clinical results (Kairamkonda et al., 2008) for health professionals when detecting respiratory illnesses.

This research has some limitations that should be acknowledged and considered for future research. Cross-sectional information was recorded for the research. This design prevented analysis and interpretation of the temporal changes in patients. In addition, it was not possible to measure some variables that could have influenced lung function, such as thoracic perimeter, physical activity, and levels of atmospheric contamination during the months of data collection. In spite of this, it is estimated that between August and December (data collection), the levels of atmospheric contamination in the Maule Region diminished considerably due to the decrease in pollutants due to household heating. Pollution is greater during the winter months of June and July.

This research also has some strengths. For example, this is the first study carried out in Chile where percentile norms are proposed to evaluate MEF based on biological age. On the other hand, quality control was maintained by evaluating measurements two times for 10% of the total student sample. Furthermore, the probabilistic selection of the sample guaranteed generalizability to contexts with characteristics similar to those in this study.

## Conclusion

In conclusion, the MEF values for the students of the Maule Region (Chile) are lower than those reported for international references. Moreover, biological age was more useful when related to chronological age. In view of this, regional percentiles have been created to facilitate the evaluation of the inspiratory muscle strength of children and adolescents based on biological age. We recommend that the norms be used to diagnose and monitor the risk of asthma and the general respiratory state of pediatric populations. The calculations can be carried out using the following link: http://www.reidebihu.net/adolescentes.php.

## Supplemental Information

10.7717/peerj.5157/supp-1 Supplemental Information 1 Reference Norms for chronological age.

10.7717/peerj.5157/supp-2 Supplemental Information 2 Reference Norms for biological age.

## Additional Information and Declarations

### Data Availability

The following information was supplied regarding data availability: Bolaños, Marco Cossio; Andruske, Cynthia Lee; de Arruda, Miguel; Torres, Jose Sulla; Carrillo, Jaime Pacheco; Urra-Albornoz, Camilo; Gomez-Campos, Rossana (2018): Reference Norms for Evaluating Maximum Expiratory Flow of Children and Adolescents of the Maule Region in Chile. figshare. Dataset. DOI 10.6084/m9.figshare.5687392.v1.
