# Supplementary figures and images for: Reference norms for evaluating maximum expiratory flow of children and adolescents of the Maule Region in Chile
Source: PeerJ. 2018 Jul 16;6:e5157. doi: 10.7717/peerj.5157 (PMC6052850; doi:10.7717/peerj.5157)

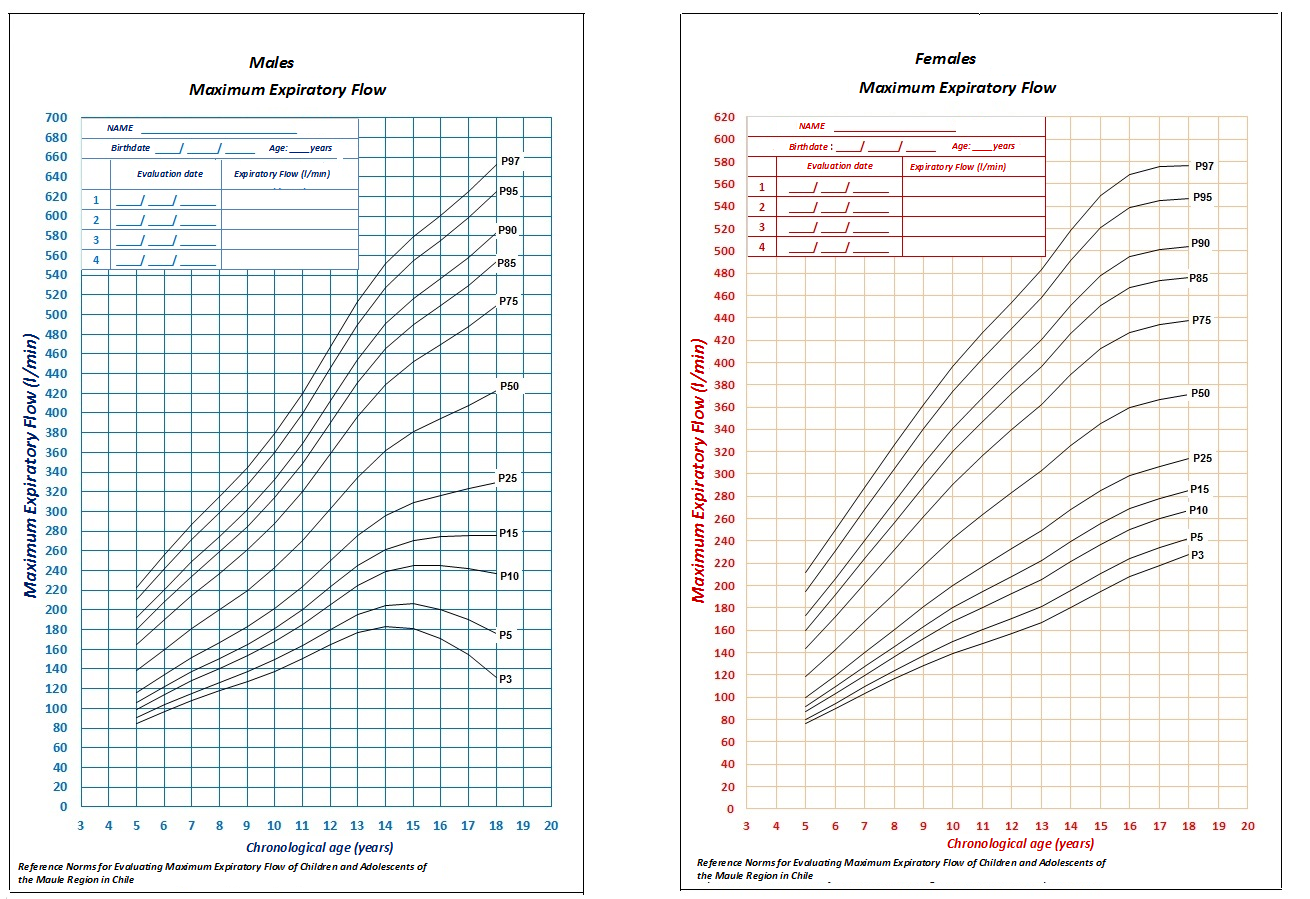

Supplement: Supplemental Information 1 [file peerj-06-5157-s001.png]

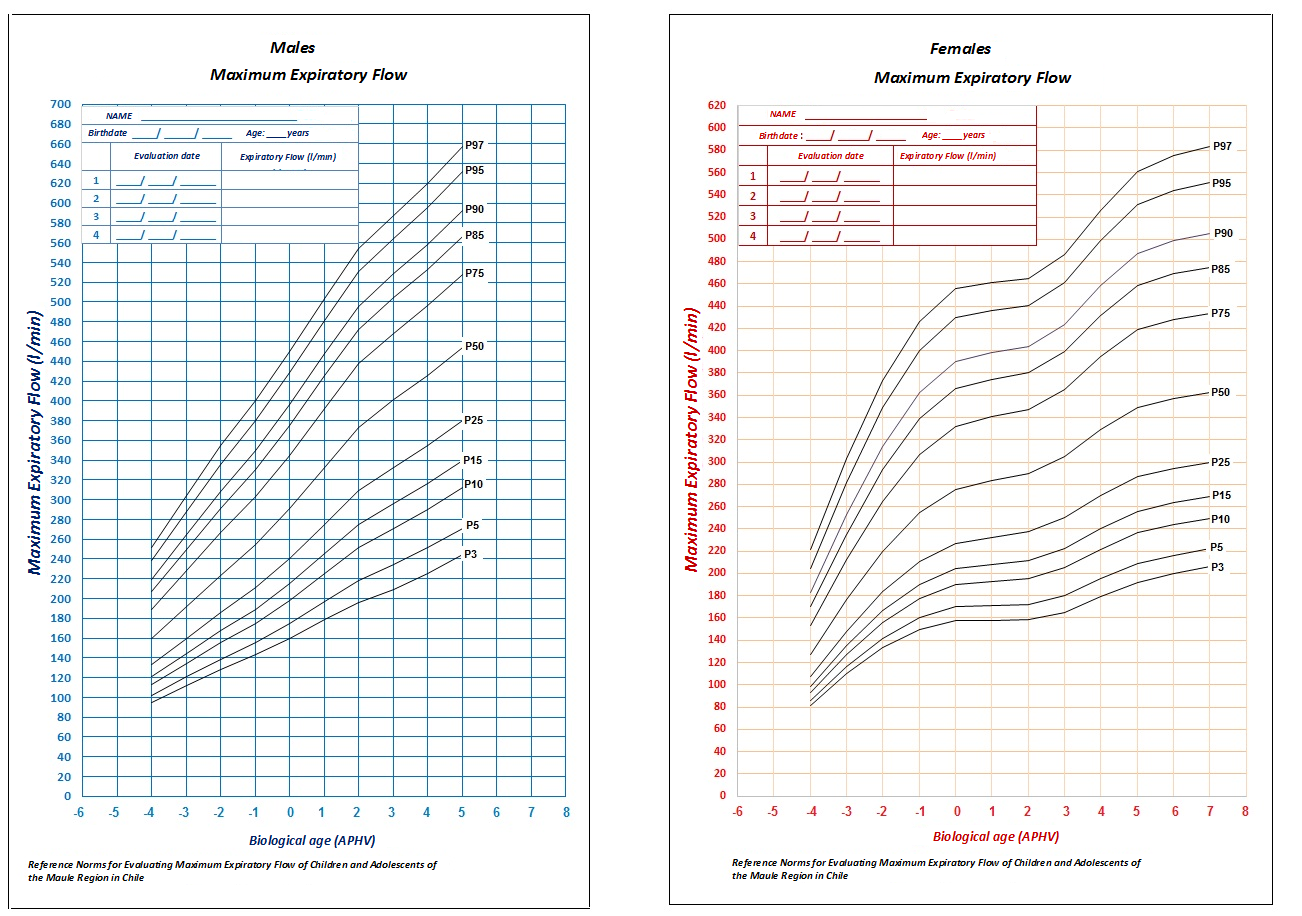

Supplement: Supplemental Information 2 [file peerj-06-5157-s002.png]
